# Poincare guided geometric UNet for left atrial epicardial adipose tissue segmentation in Dixon MRI images

**DOI:** 10.1038/s41598-025-10110-1

**Published:** 2025-07-15

**Authors:** Marjan Firouznia, Erik Ylipää, Markus Henningsson, Carl-Johan Carlhäll

**Affiliations:** 1 Unit of Cardiovascular Sciences, Department of Health, Medicine and Caring Sciences, Linköping University, Linköping, Sweden; 2 AIDA Data Hub, Linköping University, Linköping, Sweden; 3 Center for Medical Image Science and Visualization (CMIV), Linköping University, Linköping, Sweden; 4 Department of Clinical Physiology in Linköping, Department of Health, Medicine and Caring Sciences, Linköping University, Linköping, Sweden

**Keywords:** Epicadial adipose tissue, Left atrium, Convolutional neural networks, Segmentation, Poincaré layer, Deep learning, Cardiology, Diseases, Health care, Medical research, Engineering, Mathematics and computing

## Abstract

Epicardial Adipose Tissue (EAT) is a recognized risk factor for cardiovascular diseases and plays a pivotal role in the pathophysiology of Atrial Fibrillation (AF). Accurate automatic segmentation of the EAT around the Left Atrium (LA) from Magnetic Resonance Imaging (MRI) data remains challenging. While Convolutional Neural Networks excel at multi-scale feature extraction using stacked convolutions, they struggle to capture long-range self-similarity and hierarchical relationships, which are essential in medical image segmentation. In this study, we present and validate PoinUNet, a deep learning model that integrates a Poincaré embedding layer into a 3D UNet to enhance LA wall and fat segmentation from Dixon MRI data. By using hyperbolic space learning, PoinUNet captures complex LA and EAT relationships and addresses class imbalance and fat geometry challenges using a new loss function. Sixty-six participants, including forty-eight AF patients, were scanned at 1.5T. The first network identified fat regions, while the second utilized Poincaré embeddings and convolutional layers for precise segmentation, enhanced by fat fraction maps. PoinUNet achieved a Dice Similarity Coefficient of 0.87 and a Hausdorff distance of 9.42 on the test set. This performance surpasses state-of-the-art methods, providing accurate quantification of the LA wall and LA EAT.

## Introduction

Atrial Fibrillation (AF) is a common cardiac arrhythmia associated with an elevated risk of heart failure and thromboembolism, leading to increased mortality and morbidity^1^. While AF development is influenced by the enlargement of the Left Atrium (LA), LA Epicardial Adipose Tissue (EAT) also plays a significant role^2,3^. Positioned near the atrial myocardium, and also the coronary arteries, EAT acts as a metabolically active organ, impacting cardiac function and contributing to cardiovascular pathology. In the context of conditions such as AF, EAT around LA is implicated in LA remodeling, myocardial inflammation, and fibrosis. Recent studies underscore the crucial role of EAT in AF pathophysiology, revealing its intricate association with LA structural changes and elevated susceptibility to arrhythmias^4^.

Magnetic Resonance Imaging (MRI) has been used to visualize the extent and distribution of EAT^5^. The segmentation and quantification of LA and EAT from Dixon fat–water separated MRI data can provide reliable information for risk stratification and clinical management^6^. Automated segmentation can overcome the limitations of manual processing (time-consuming and operator dependent). Challenges of automation encompass issues such as suboptimal image quality, diverse LA shapes, and thin LA walls. Moreover, the variation in size, shape, and position of EAT between the heart and pericardium introduces additional complexities^7^.

Numerous deep learning methods address LA segmentation in MRI, including UNet^8^, bidirectional convolutional LSTM^9^, and 3D attention UNet^10^. Daudé presented a Deep-learning segmentation method for EAT using four-chamber Late Gadolinium Enhancement (LGE) MRI^11^. Langner et al.^12^ applied UNet and VNet for automated segmentation of visceral and subcutaneous adipose tissue in Dixon water–fat MRI data. Additionally, Estrada et al. introduced FatSegNet, a deep learning pipeline for precise abdominal adipose tissue segmentation in Dixon MRI, demonstrating high accuracy and reliability^13^. FM-Net, a fully automatic double ResUNet method, also tackled EAT segmentation using fat maps from Dixon MRI^14^. However, traditional methods still struggle with the complex hierarchical structure of EAT, especially around the LA, due to issues like low intensity contrast, inhomogeneous signals, and class imbalance.

Hierarchical relationships and the organization of fat pixels and surrounding tissues around the LA can be demonstrated through anatomical studies that reveal nested structures. Traditional CNN-based methods, including UNet variants, often miss small terminal branches and suffer from global information loss due to limited contextual awareness. Poincaré embeddings, inspired by hyperbolic neural networks ability to model structural information with fewer parameters, offer a solution. By capturing hierarchical structures more effectively, Poincaré layers improve segmentation accuracy and preserve anatomical hierarchies better than conventional convolutional layers, providing robust evidence for their efficacy in complex medical image segmentation tasks^15^.

Recently, hyperbolic embeddings have gained prominence in machine learning, showcasing their ability to more accurately represent hierarchical data compared to Euclidean space using Poincaré maps^15^. In this study, the potential of Poincaré embedding is leveraged to introduce a novel framework based on deep neural networks, termed PoinUNet. This framework is designed for the joint segmentation and quantification of the blood cavity and EAT of LA from LGE Dixon images. The methodology revolves around the development of a specialized 3D UNet variant, incorporating Poincaré layers to extract crucial geometric insights for multi-label segmentation. PoinUNet integrates a Poincaré layer into its convolutional layer architecture, merging Euclidean and hyperbolic feature spaces. This enhances UNet segmentation capabilities by capturing global structural context through local information in convolutional layers.

Accordingly, the aim of this study is to present and validate an automated Poincaré-guided geometric UNet (PoinUNet) method for quantifying the LA wall and the EAT around the LA from LGE Dixon MRI data.

## Methodology

**Fig. 1:**
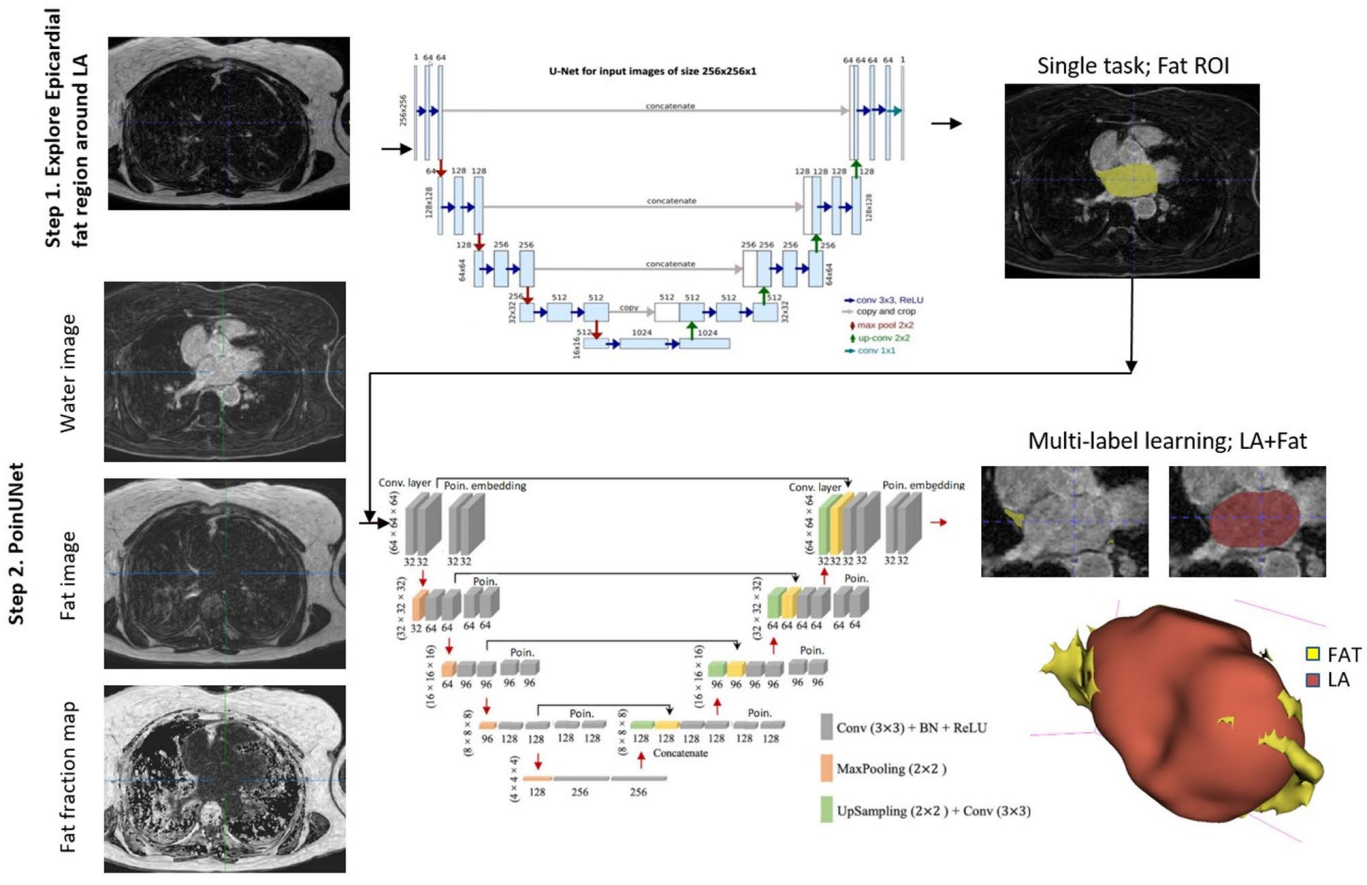
The proposed PoinUNet model for segmentation of the left atrium (LA) and the surrounding epicardial adipose tissue (EAT) by using water and fat images and fat fraction maps obtained from Dixon MRI sequences. The pipeline comprises two main steps: (Step1) Training a 3D UNet for finding a region of interest selection to actually crop out the predicted region and the feed only that to the step 2 network using fat images as the input; (Step 2) Training a PoinUNet for joint segmentation of EAT and LA. This involves a multi-label segmentation approach with three input channels: water, fat, and fat fraction maps.

The PoinUNet method employs a deep learning architecture integrating convolutional and Poincaré layers of 3D UNet for the automated segmentation of LA and the surrounding epicardial fat. This segmentation is accomplished by utilizing four input images to feed the network. The proposed method is a fully automatic segmentation, and an overview of the proposed method is shown in Fig. 1.

While UNet uses an encoder-decoder structure to reduce input dimensions and capture features through convolution and downsampling, PoinUNet enhances this by introducing hyperbolic space for better hierarchical feature extraction. The model consists of two stages: in the first stage, the model is trained on fat images to distinguish fat regions and enhance anatomical hierarchies. The second stage refines segmentation predictions using Poincaré-based layers and is trained with three input images—fat, water, and fat fraction maps. This approach shifts features from Euclidean to hyperbolic space, allowing the model to effectively analyze the relationship between EAT and LA and to separate LA fat from EAT in other organs using these three image features.

Moreover, PoinUNet employs Hyperbolic Multinomial Logistic Regression (HMLR) to compute the cross-entropy loss function and performs gradient descent, ensuring accurate segmentation, particularly for 3D image tasks. The model optimizes the loss function using balanced weighted coefficients, thereby providing a comprehensive approach to pixel-wise classification and segmentation.

### Clinical MRI data

This prospective study was approved by the Swedish Ethical Review Authority. All participants provided written informed consent to participate. We confirm that the study was performed in accordance with relevant guidelines and regulations, including the Declaration of Helsinki.

The study comprised a total of 66 participants with a clinical indication for cardiac MRI. Specifically, the cohort consisted of 48 subjects with a history of AF, 9 subjects with other cardiac diseases, and 9 subjects without a history of cardiac disease. The MRI scans were performed using a 1.5T MRI scanner (Philips Healthcare, Best, The Netherlands) equipped with 28-channel receive coils. The LGE Dixon scans had a field-of-view (FOV) of 320 × 320 × 120–140 mm^3^, and a spatial resolution of 1.25 × 1.25 × 2.5 mm^3^.

### Manual labeling and preprocessing

In the methodology, a hybrid approach is employed, combining manual segmentation with automated pixel editing for the precise delineation of EAT around LA in the MRI images. To validate the approach, the threshold-based method introduced by Skoda et al.^5^ is utilized as the ground truth.

**Fig. 2:**
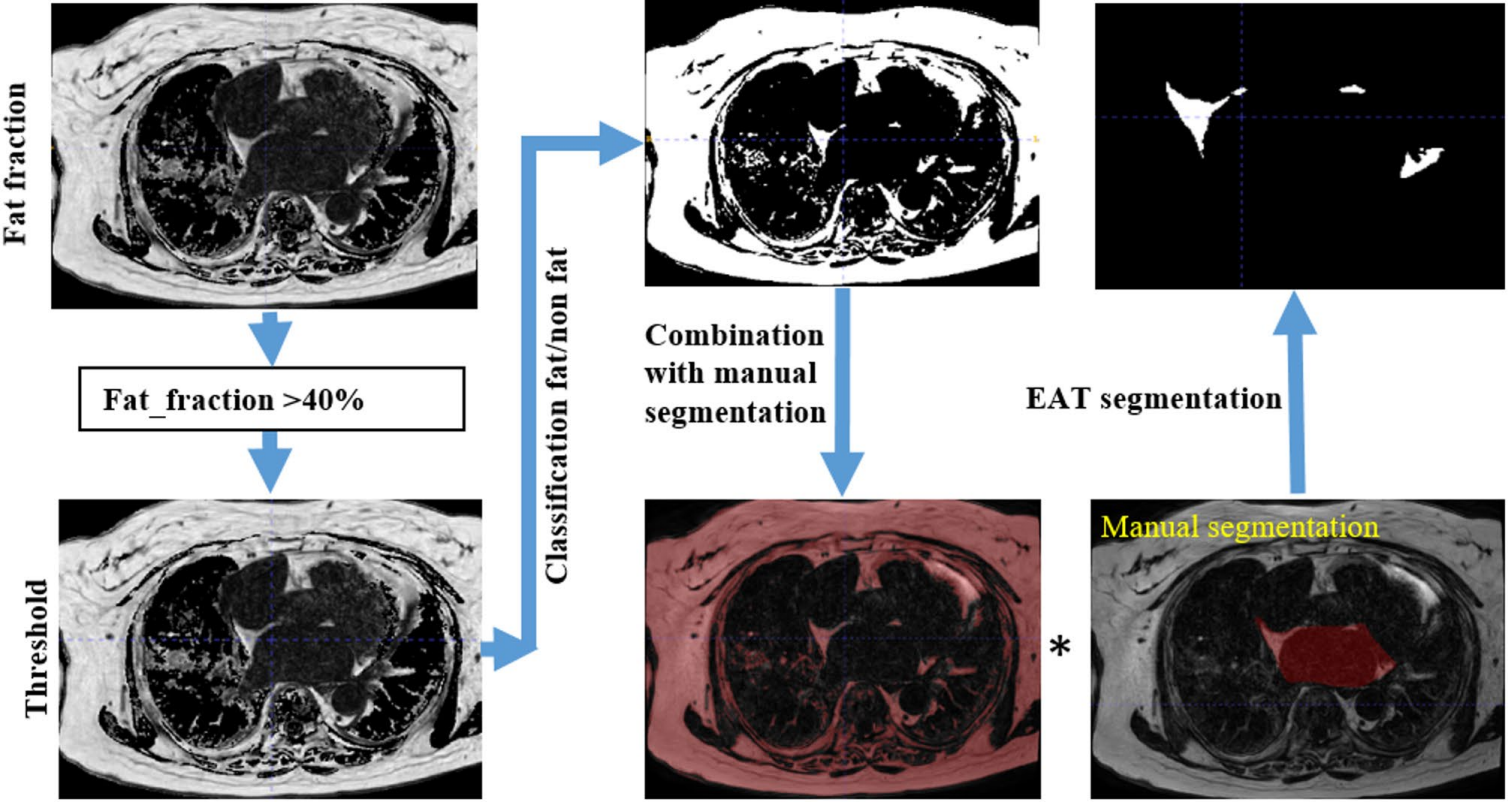
EAT segmentation ground truth obtained through semi-automatic segmentation. A schematic illustration of the proposed pipeline: starting with the source fat image and fat fraction map, followed by classification using thresholding, and concluding with the final segmentation by combining the classified output with manual fat ROI segmentation.

To distinguish between fat and non-fat signals in each slice, the thresholding process involves applying a fat fraction threshold of 0.4 to the fat fraction map (Fig. 2). Voxels exhibiting fat fractions exceeding 0.4 in the normalized fat fraction map are selectively retained, effectively removing pixels in the LA blood pool and contributing to the delineation of the EAT region (Fig. 2). This approach ensures accurate identification of EAT regions based on relative fat content, thereby enhancing the segmentation process. Multiplying the output with the manual segmentation of fat provides references for accurate identification and validation purposes^5^.

### PoinUNet: poincaré embedding learning for segmentation

In this study, a novel approach is introduced to simultaneously segment EAT and LA from LGE Dixon MRI images. In the first stage (step 1), a traditional 3D UNet extracts features and performs initial segmentation, distinguishing LA fat from non-fat regions and fat in other organs. The second stage (step 2) refines this segmentation by combining a similar 3D UNet with Poincaré-based layers, which preserve local features and relational consistency during decoding. The model generates two feature maps, ${F}_{e}$ for feature map in Euclidean space and ${F}_{h}$ for feature map in Poincaré embedding or hyperbolic space, It leverages an attention mechanism to compute edge weights between nodes in the hyperbolic relational space, using significant local patterns and convolutional operations to enhance segmentation performance.

The dataset of fat images is denoted as $X=\left\{{x}_{i}:i=1,\dots ,N\right\}$, where ${x}_{i}$ has dimensions D*W*H. The corresponding ground-truth labels are denoted as $\left\{{y}_{i}\right\}$ , where each ${y}_{i}$ is a three-class label including background, LA, and EAT. The encoder section employs Conv3D layers for spatial feature extraction as ${F}_{e}$. The convolutional operation is defined as:

1 $$Conv3D\left( {X,W_{c} ,b} \right) = \sigma \left( {X*W_{c} + b} \right)$$

where X is the input tensor, W represents filters, b denotes biases, and *σ* is the non-linear ReLU activation function. Following Conv3D layers, the Poincaré embedding layer is introduced to map Euclidean space features into hyperbolic space, enabling the model to capture hierarchical relationships inherent in medical imaging data.

#### Poincaré embedding layer

**Fig. 3:**
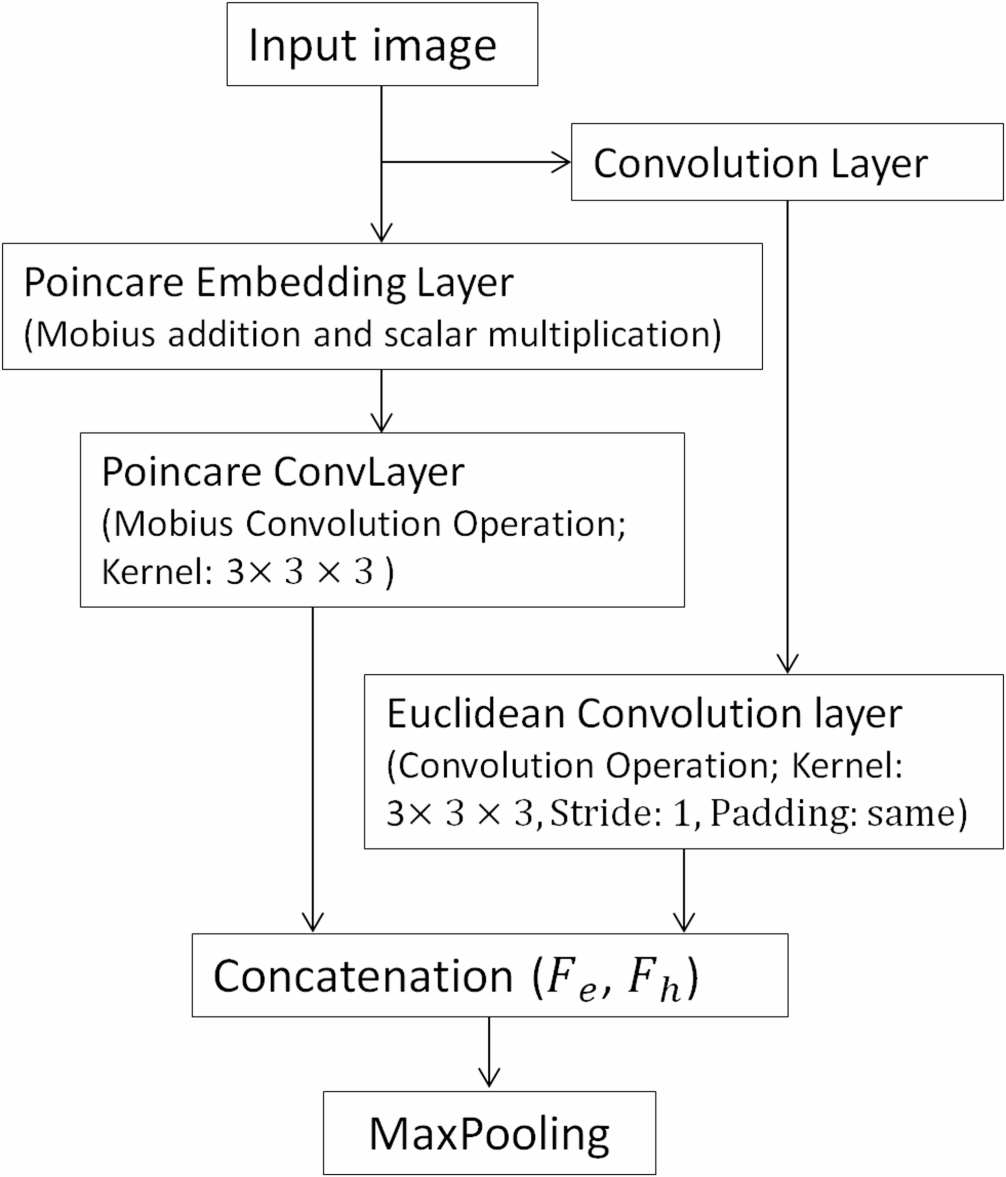
Encoder architecture of PoinUNet; The input image is processed through a convolution layer, followed by parallel hyperbolic (Poincaré embedding and Möbius convolution) and Euclidean convolution layers. The resulting feature maps (${F}_{h}$ and ${F}_{e}$) are concatenated and downsampled using MaxPooling for further processing in the decoder.

PoinUNet uses the Poincaré embedding layer (Fig. 3), which maps features from Euclidean space into hyperbolic space. This layer captures hierarchical relationships within medical imaging data, denoted as ${F}_{H}$. In this model, image pixels are transformed to their hyperbolic equivalents. The Poincaré ball of curvature $k$, with a Riemannian manifold is defined as

2 $$g_{P}^{{{\mathcal{H}}_{k}^{n} }} = \left\{ {P \in {\mathbb{R}}^{n} :k\parallel P\parallel^{2} < 1, \quad k \ge 0} \right\},$$

the Riemann metric $\lambda_{P}^{{{\mathcal{H}}_{k}^{n} }}$ represents the scaling factor, given by $\lambda_{P}^{{{\mathcal{H}}_{k}^{n} }} = \frac{2}{{1 - k\parallel P\parallel^{2} }}$ ,and $g^{ \in } = I^{n}$ denotes the Euclidean metric.

#### Hyperbolic operations

The Poincaré ball model effectively represents shape and curvature without considering size or position, making it useful for complex datasets. Its application in machine learning is increasing, with Chine et al.^16^ using it for classification, and Guo et al.^17^ proposing a Poincaré-based neural network for sequential recommendation. This network captures both sequential patterns and hierarchical relationships, adapting basic neural network operations to hyperbolic geometry^18^.

- *Möbius Addition* $\oplus$ is given by
  3 $$q_{1} \oplus q_{2} = \frac{{1 + 2k\langle q_{1} ,q_{2}\rangle + k\parallel q_{2}\parallel^{2} }}{{1 + 2k\langle q_{1} ,q_{2}\rangle + k^{2} \parallel q_{1}\parallel^{2}  q_{2}\parallel^{2} }}q_{1} + \frac{{1 - k^{2} \parallel q_{1}\parallel^{2} }}{{1 + 2k\langle q_{1} ,q_{2}\rangle + k^{2} \parallel q_{1}\parallel^{2} \parallel q_{2}\parallel^{2} }}q_{2} .$$
  here ${q}_{1}$ and ${q}_{2}$ are vectors in hyperbolic space.
- *Möbius Scalar Multiplication:*
  4 $$s \otimes q = \frac{1}{\sqrt k }\tanh \left( {q.tanh^{ - 1} \left( {\sqrt k \parallel x\parallel} \right)} \right)\frac{x}{\left\| x \right\|}$$
  here s is a scalar and $q$ is a vector in hyperbolic space.

#### Transformations between spaces

To segment images, a mapping from Euclidean to hyperbolic space using two maps is necessary. To transform a vector $x$ in Euclidean space to a vector $q$ in hyperbolic space, the exponential map is used:

5 $$exp_{P}^{{{\mathcal{H}}_{k}^{n} }} \left( x \right) = P \oplus \left( {\tanh \left( {\frac{\sqrt k \left\| x \right\|}{{2\lambda_{P}^{{{\mathcal{H}}_{k}^{n} }} }}} \right)\frac{x}{\sqrt k \left\| x \right\|}} \right)$$

here $\oplus$ represents Möbius addition, and $P$ is the point of origin in the hyperbolic space. To transform a vector $q$ in hyperbolic space to a vector $x$ in Euclidean space, the logarithmic map is computed as

6 $$log_{P}^{{{\mathcal{H}}_{k}^{n} }} \left( q \right) = \left( {\frac{{2\sqrt k \lambda_{P}^{{{\mathcal{H}}_{k}^{n} }} }}{{tanh^{ - 1} \left( {\sqrt k \left\| { - P \oplus q} \right\|} \right)}}} \right) - \left( {P \oplus q} \right)\frac{1}{{\left\| { - P \oplus q} \right\|}}$$

For image segmentation, PoinUNet employs HMLR, which leverages the hyperbolic distance between points and predefined class planes to classify each voxel. The hyperbolic distance between the point ${z}_{ij}$ and the plane ${g}_{P}^{{\mathcal{H}}_{k}^{n}}$ of class $y$ is computed as:

7 $$d\left( {z_{ij} ,g_{P}^{{{\mathcal{H}}_{k}^{n} }} } \right) = \frac{1}{\sqrt k }sinh^{ - 1} \left( {\frac{{2\sqrt k \left\langle {p_{y} \oplus z_{ij} ,a_{y} } \right\rangle }}{{\left( {1 - k\left\| {p_{y} \oplus z_{ij} } \right\|^{2} } \right)\left\| {a_{y} } \right\|}}} \right)$$

where $a$ is the normal vector of plane^19–21^.

HMLR operates entirely in hyperbolic space, computing distances using Möbius operations and hyperbolic distance metrics. Möbius Convolution, performed in hyperbolic space with Möbius addition and scalar multiplication, is approximated to reduce memory and computational overhead, allowing efficient calculation of logits for each pixel. The logit calculation is given by:

8 $$\hat{y} \propto exp\left( {\zeta y\left\langle {zij} \right\rangle } \right)$$

$\hat{y}$ represents the logit value of the pixel, $y$ is the ground truth label of the pixel, $zij$ denotes the hyperbolic representation of the pixel, ζ is a parameter for scaling or weighting the inner product operation, ⟨⟩ represents the inner product operation between the hyperbolic representations of the pixel and the ground truth label. HMLR also computes the cross-entropy loss, with the parameter $k$ (defining the Poincaré ball size) playing a significant role in segmentation performance. Varying $k$ affects the feature distribution in hyperbolic space, and the value of $k$ is trained using exponential parametrization:

9 $$k = e^{{l_{k} .{\sigma }}} + l_{0}$$

where ${l}_{k}$ serves as a hyperparameter controlling the scaling of $k$ based on the value of $\upsigma$, while ${l}_{0}={10}^{-3}$ adjusts the base value^22^.

To further refine the approach, an Inverse Distance Weighted (IDWH) loss is used^23^. The IDWH loss prioritizes voxels near LA, allowing for the segmentation of finer structures and safeguarding the EAT region, all while excluding consideration for EAT from other organs. The IDWH loss function focuses on segmenting voxels near the LA to preserve fine structures and protect the EAT region, excluding EAT from other organs. Voxels closer to the LA boundary receive higher weights. Let ${V}_{LA}$ be the set of voxels in the LA region, and $d(v)$ the distance from voxel $v$ to the LA boundary. The IDWH loss integrates the inverse distance weight map into the Dice loss, and is defined as:

10 $$L_{IDWH} = 1 - {{\left( {\mathop \sum \limits_{c = 1}^{C} \frac{{2\mathop \sum \nolimits_{k = 1}^{K} p_{k,c} y_{k,c} \cdot d_{k} }}{{\mathop \sum \nolimits_{k = 1}^{K} p_{k,c}^{2} \cdot d_{k} + \mathop \sum \nolimits_{k = 1}^{K} y_{k,c}^{2} .d_{k} }}} \right)} \mathord{\left/ {\vphantom {{\left( {\mathop \sum \limits_{c = 1}^{C} \frac{{2\mathop \sum \nolimits_{k = 1}^{K} p_{k,c} y_{k,c} \cdot d_{k} }}{{\mathop \sum \nolimits_{k = 1}^{K} p_{k,c}^{2} \cdot d_{k} + \mathop \sum \nolimits_{k = 1}^{K} y_{k,c}^{2} .d_{k} }}} \right)} C}} \right. \kern-0pt} C}$$

where $c \in C$ denotes $c$ th class, $k \in K$ represents $k$ th voxel in the image, *p* ∈ *P* means predictions by the network, and $d \in DF$ are the inverse distance weights^23^.

The approach enhances pixel-wise classification by combining hyperbolic distances, logit cross-entropy, adaptive curvature, and Euclidean weights. These components are integrated into a comprehensive loss function, optimizing segmentation tasks through the unique properties of hyperbolic space and adaptive weighting strategies.

11 $$\mathcal{L}=\alpha \times {L}_{cross\_entropy }+\gamma \times {L}_{Curvature}(k)+\delta \times {L}_{IDWH}+\varepsilon * {L}_{Euclidean\_Weight\text{s}}$$

where $\upalpha = 0.5$, γ = 0.2, δ = 0.5, ${\upvarepsilon } = 0.2$ are weighted coefficients. In Fig. 3 shows the Poincare embedding layer to training the model based on EAT and LA segmentation.

In hyperbolic network embedding for pixel segmentation, the model uses a Poincaré embedding layer to transform pixel features from Euclidean to hyperbolic space using Möbius addition and scalar multiplication, inspired by Möbius matrix–vector multiplication as described by Liu et al.^20^ This transformation captures intricate spatial and hierarchical relationships within the image. The UNet architecture processes data in parallel through both hyperbolic and Euclidean spaces: hyperbolic convolutional layers extract geometric and structural information, while Euclidean convolutional layers handle pixel-level features from the original image. This dual processing enhances pixel-wise classification accuracy. The values for the weighted coefficients were chosen through empirical evaluation. They were selected to balance the contributions of different loss components based on their importance in the segmentation task. While these values may vary depending on the architecture and dataset characteristics, they were chosen consistently across all compared architectures for fair evaluation.

### Comparative analysis

Two algorithms were are used for comparison and ablation studies. The first one was the baseline algorithm that used a 3D UNet architecture for LA segmentation^11^. The MONAI library, a PyTorch-based framework for medical imaging, was used to implement a 3D UNet with adjustments to filters and layers for EAT segmentation. Data augmentation techniques like random rotation, scaling, and flipping are applied to enhance model robustness and prevent overfitting. All operators in these encoder and decoder were the same as those specified in Fig. 1. The second algorithm, SwinUNETR, also used a multi-label network, with the same encoder and decoder architectures shown in original reference^24,25^.

SwinUNETR is specifically adapted for 3D segmentation, with modifications to augmentations, patch size, and parameters, whereas SwinUNet, a 2D segmentation model, processes individual 2D slices without considering explicit volumetric context. For SwinUNETR, non-overlapping 96 × 96 × 96 patches are projected into embedding tokens, encoded by a 3D Swin Transformer with self-attention in local windows and interaction via 3D window shifting. The encoder partitions tokens into 3D embeddings, using a 2 × 2 × 2 patch size and 48 embedding dimensions. It has four stages, each with two transformer blocks, and applies patch merging to reduce resolution by 2. A CNN decoder, connected via skip connections, processes the reshaped features with 3 × 3 × 3 residual blocks, upsamples by 2, and outputs the segmentation via a 1 × 1 × 1 convolution with sigmoid activation. The voxel size in the 3D UNet typically represents a 96 × 96 × 96 grid of voxels.

All experiments were conducted using 18/36 cores/threads, 256 GB RAM, and an RTX 4070Ti 12GB GPU. The dataset was initially split into training (54 individuals), validation (6 individuals), and testing (6 individuals) subsets. This initial split was used for hyperparameter tuning and determining the number of iterations for training. Based on this, a fixed number of training iterations was determined, which was then used consistently across all experiments. To ensure the robustness of the proposed PoinUNet model, cross-validation experiments were conducted to enhance the reliability and generalizability of the results. Given the limited dataset of only 66 individuals, cross-validation was essential to mitigate the risk of overfitting and provide more reliable insights into model performance.

The model underwent training for 600 epochs with a batch size of 2. The optimization of Euclidean parameters employed stochastic gradient descent with a momentum of 0.9 and polynomial learning rate decay with a power of 0.9. Hyperbolic parameters were optimized using Riemannian stochastic gradient descent^19^. Poincaré layers were configured with a maximum of 10,000 iterations and a learning rate of 0.1.

The standard error of the mean (SEM) reflects the variability of the model’s performance across the 15 runs (3 folds × 5 runs for each fold). Specifically, SEM is calculated from the performance scores of the models for each test subject within a fold. It shows how the average performance of each model deviates from the overall mean performance across all 15 runs. This ensures that the reported performance is stable and representative of how the model generalizes across different subsets of the dataset. Clinical assessment of EAT and LA volume was conducted to ensure the results applicability in clinical settings. This involved comparing mean volume values and using correlation and Bland–Altman plots. Additionally, comparisons were made to evaluate the method dependency on the type of fibrillation, age, and class imbalance in PoinUNet, with an analysis of different loss functions used in the model.

#### Evaluation metrics

In this study, various evaluation metrics were employed to comprehensively assess the accuracy of the estimated EAT segmentation compared to the ground truth. The Intersection over Union (IoU) percentage quantified the overlap between the predicted and ground truth segmentations, while the Dice Similarity Coefficient provided a measure of their similarity. Precision evaluated the model ability to identify positive instances accurately, while recall assessed its capacity to capture all positive instances from the ground truth. The F1-score served as a balanced measure of accuracy, considering both false positives and false negatives. Additionally, the Hausdorff Distance (HD) and Average Symmetric Surface Distance (ASD) measured the spatial accuracy and overall agreement between the predicted and ground truth segmentations, respectively.

### Statistical analysis

Resuls are given as group Mean ± SEM and a *P* < 0.05 was considered significant. Statistical analysis was performed on EAT and LA volumes, and segmentation metrics (Dice, IoU, Precision, Recall, F1-score, HD, and ASD) using methods like PoinUNet, 3D UNet, and SwinUNETR. Normality was assessed using the Shapiro–Wilk test, and most of the data were found to be normally distributed. For normally distributed data, one-way ANOVA with Tukey’s post-hoc test was used, while for non-normally distributed data, Kruskal–Wallis with Wilcoxon post-hoc tests were applied. For these tests the adjusted P-values are reported. To evaluate the dependency of EAT and LA volumes on AF type and age, *t*-tests or Wilcoxon tests with Bonferroni correction for multiple comparisons (correction factor 8) were used. Statistical analysis was performed using IBM SPSS Statistics for Windows, version 26.0 (IBM Corp., Armonk, N.Y., USA).

## Results

### Validation of PoinUNet on test set

The performance of PoinUNet was evaluated by comparing this model to state-of-the-arts using manual segmentations of LA and EAT conducted by expert readers. PoinUNet showed promising results in comparison to SwinUNETR and 3D UNet with a Dice score of 0.87 on 6 MRI data sets. In assessing the segmentation performance across various tasks, the Dice scores reported in Table 1 serve as indicators. PoinUNet consistently outperforms other models, especially when equipped with three input channels (water, fat, and fat fraction map). For the LA (single task), EAT (single task), and multi-label segmentation, PoinUNet achieves Dice scores of 0.94, 0.75, and 0.87, respectively.

**Table 1:** Summary of Dice scores for different deep learning models in both single and multi-label segmentation tasks.

	SwinUNETR (water and fat)	SwinUNETR (water and fat and fat fraction map)	3D UNet (water and fat)	3D UNet (water and fat and fat fraction map)	PoinUNet (water and fat)	PoinUNet (water and fat and fat fraction map)
LA (single task)	91.12 ± 0.02	91.49 ± 0.11	90.01 ± 0.45	90.15 ± 0.21	93.74 ± 0.76	93.55 ± 0.32
EAT (single task)	69.23 ± 0.52	74.64 ± 0.16	44.94 ± 1.43	56.51 ± 0.92	72.31 ± 0.40	75.45 ± 0.59
Multi label segmentation	76.32 ± 0.67	80.21 ± 0.68	67.60 ± 0.86	67.83 ± 1.54	81.83 ± 0.41	87.25 ± 0.49

The average Dice scores obtained on a 6-individual test set.

The intervals reported in Table 2 represent the spread of performance metrics over all segmentations within test set. A comprehensive comparison of segmentation performance between our proposed method (PoinUNet), 3D UNet, and SwinUNETR is presented, specifically focusing on LA and EAT segmentation with three input channels. PoinUNet demonstrates better performance across multiple evaluation metrics. PoinUNet demonstrates consistently higher IoU, Dice scores, and recall than both UNet and SwinUNETR, while achieving lower HD. In Table 2, these metrics were calculated for each image separately and averaged over all test set images that contain at least one ROI in their ground truths. The SEM indicates that the model performance is stable across the test set, with PoinUNet (using three input channels) exhibiting minimal variation in results compared to other models. Overall, these results demonstrate that PoinUNet provides significantly superior performance for both EAT and LA segmentation compared to 3D UNet and SwinUNETR.

**Table 2:** Comparison of the proposed PoinUNet method with 3D UNet and SwinUNETR methods, using three channels for LA and EAT segmentation.

	3D UNet	SwinUNETR	PoinUNet
IoU (%)↑	68.25 ± 1.78***	74.63 ± 0.68***	78.61 ± 0.50
Dice (%)↑	67.83 ± 1.54***	80.21 ± 0.68***	87.25 ± 0.49
Precision (%)↑	79.83 ± 1.53***	84.82 ± 1.06	88.61 ± 0.55
Recall (%)↑	79.16 ± 1.19***	82.42 ± 0.56*	88.10 ± 0.66
F1-score (%)↑	80.46 ± 1.31***	85.02 ± 0.69	88.33 ± 0.30
HD↓	24.35 ± 1.60***	13.68 ± 0.74***	9.42 ± 0.54
ASD↓	7.51 ± 0.66***	4.11 ± 0.31	2.94 ± 0.17

The test set validation results are based on six individuals, with average scores calculated across all slices in the test set.

↑higher is better, ↓lower is better. Significant difference from PoinUNet: *P < 0.005, ***P < 0.0001.

**Fig. 4:**
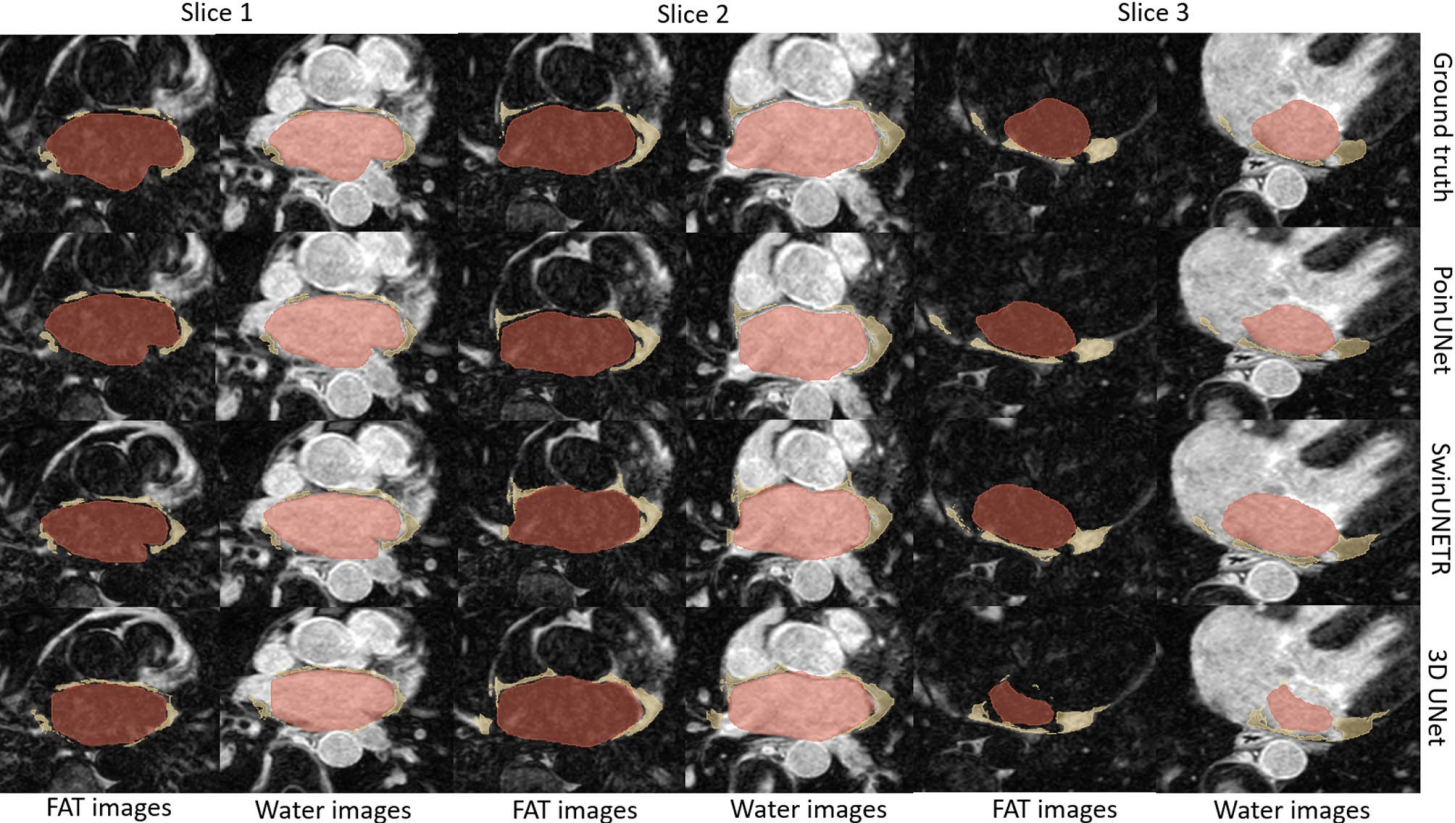
Visual comparison of segmentation results for different methods. Each row represents the segmentation results for each method (Ground truth, PoinUNet, SwinUNETR, 3D UNet). The red and yellow regions represent the LA and EAT segmentation results, respectively. Three same slices from one test individual.

Figure 4 demonstrates the results and performance attained by PoinUNet for multiclass segmentation, thereby emphasizing the effectiveness of Poincaré embedding layer. The visual comparisons were chosen to illustrate the challenges of accurately segmenting EAT and LA within complex anatomical contexts.

### Cross validation of PoinUNet

The cross-validation test set results in Table 3 demonstrate the performance of PoinUNet, 3D UNet, and SwinUNETR across key metrics—Precision, Recall, Dice Index, and ASD. PoinUNet achieves the highest Precision at 87.5 ± 1.7%, outperforming SwinUNETR (80.2 ± 1.9%) and 3D UNet (79.1 ± 2.9%), indicating fewer false positives. Its Recall is slightly lower at 82.2 ± 2.2% compared to SwinUNETR (86.4 ± 2.8%) and 3D UNet (84.9 ± 4.3%). However, this trade-off in recall is offset by PoinUNet superior Dice Index of 81.3 ± 1.1%, which is higher than SwinUNETR (80.6 ± 2.3%) and 3D UNet (79.0 ± 2.8%), demonstrating better overall segmentation quality. Most notably, PoinUNet achieves the lowest ASD value at 11.7 ± 1.6, surpassing both SwinUNETR (15.3 ± 2.7) and 3D UNet (15.1 ± 4.0), which indicates more accurate boundary matching.

**Table 3:** Cross-validation test set results obtained by the proposed PoinUNet models and the comparison algorithms.

Model	Precision (%)	Recall (%)	Dice Index (%)	ASD
PoinUNet	87.5 ± 0.44	82.2 ± 0.57	81.3 ± 0.28	11.7 ± 0.41
3D UNet	79.1 ± 0.75	84.9 ± 1.11	79.0 ± 0.72	15.1 ± 1.03
SwinUNETR	80.2 ± 0.49	86.4 ± 0.72	80.6 ± 0.59	15.3 ± 0.70

These are the average scores and SEM across 15 runs (three folds and five runs for each fold).

### Parameter studies and complexity

**Fig. 5:**
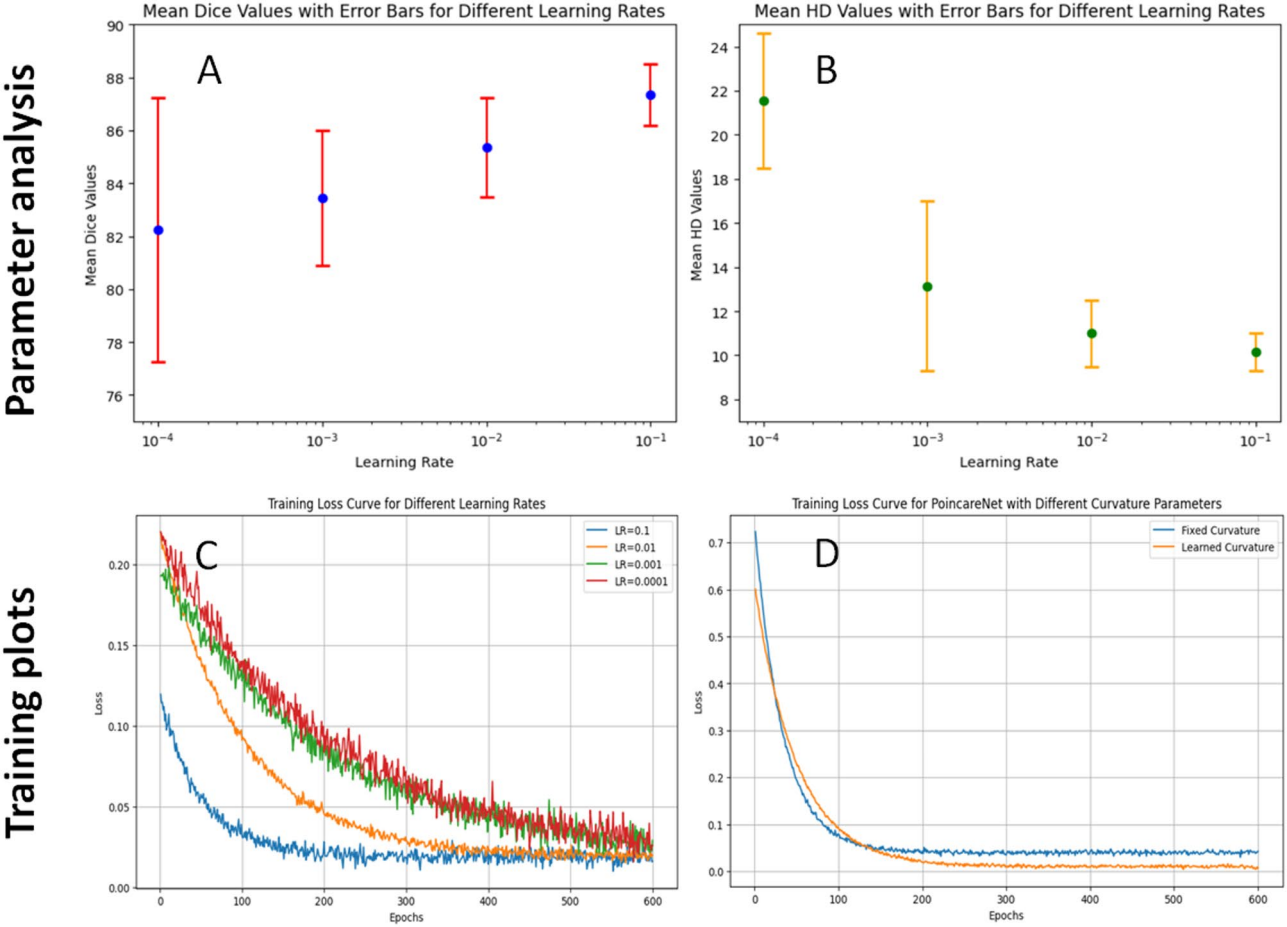
Parameter analysis (**A**, **B**); Dual panels illustrating mean Dice (**A**) and HD (**B**) values of PoinUNet for LA and EAT segmentation across varied learning rates. Training plots (**C**, **D**): Training loss curves over 600 epochs for different learning rates (LR = 0.1, 0.01, 0.001, 0.0001) (**C**), Training loss curves for fixed curvature ${10}^{-3}$ and learned curvature (**D**).

To investigate the effectiveness of the Poincaré layer, the outcomes of the proposed approach for LA and EAT segmentation were examined using various learning rates. Figure 5 shows a comparative analysis of the impact of diverse learning rates on mean Dice and HD values. This analysis indicates that a learning rate of 0.1 provides the best performance by achieving the lowest and most stable loss throughout the training epochs. The A panel showcases mean Dice values, ranging from 87.25% at a learning rate of 0.1 to 83.05% at 0.0001, with corresponding error bars denoting standard deviations. Simultaneously, the B panel provides a parallel display of mean HD values. The optimal segmentation learning rate, discerned from these results, is 0.1, associated with the highest mean Dice value (87.25%) and the lowest mean Hausdorff Distance (9.42%).

The learning rate of 0.1 demonstrates the most efficient learning with the fastest and steadiest decrease in loss, achieving the lowest final loss value (Fig. 5C). The learned curvature shows the efficient training performance in Fig. 5D.

The 3D UNet is widely recognized for its effectiveness with a moderate parameter count of approximately 34–36 million. In contrast, SwinUNETR introduces a transformer-based self-attention mechanism that dramatically increases the parameter count to around 72–83 million, enabling more nuanced feature extraction through hierarchical processing. PoinUNet, which integrates hyperbolic space transformations with a traditional convolutional backbone, strikes a balance between complexity and efficiency, resulting in a parameter count of approximately 35–40 million.

### Clinical assessment EAT and LA volumes

In this section, the EAT and LA volume measurements are compared with the ground truth and state-of-the-art methods for clinical evaluation (Table 4).

**Table 4:** Comparison of mean volumes for EAT and LA segmentation across Ground Truth and different methods.

Methods	Ground truth		3D UNet		SwinUNETR		PoinUNet	
Clinical meaurements	EAT	LA	EAT	LA	EAT	LA	EAT	LA
Mean volumes (mL)	14.70 ± 1.44	149.84 ± 7.11	16.01 ± 1.43*•	152.24 ± 7.15*•	15.85 ± 1.42*•	150.01 ± 7.14•	14.63 ± 1.35	149.02 ± 6.92

Significant difference from Ground truth: *P < 0.05. Significant difference from PoinUNet: **•***P* < 0.05.

PoinUNet demonstrates the highest accuracy in segmenting both EAT and LA volumes, with mean values closely aligning with the ground truth, indicating minimal under-segmentation. In contrast, SwinUNETR and 3D UNet show over-segmentation of EAT volumes and slight over-segmentation of LA volumes, with 3D UNet exhibiting the largest deviations compared to ground truth.

Although SwinUNETR demonstrates reasonable volume estimation, it introduces segmentation mismatches, including regions unrelated to the LA. In contrast, PoinUNet achieves more accurate anatomical delineation, with post-hoc pairwise comparisons revealing no statistically significant differences in EAT or LA volumes between PoinUNet and the ground truth.

**Fig. 6:**
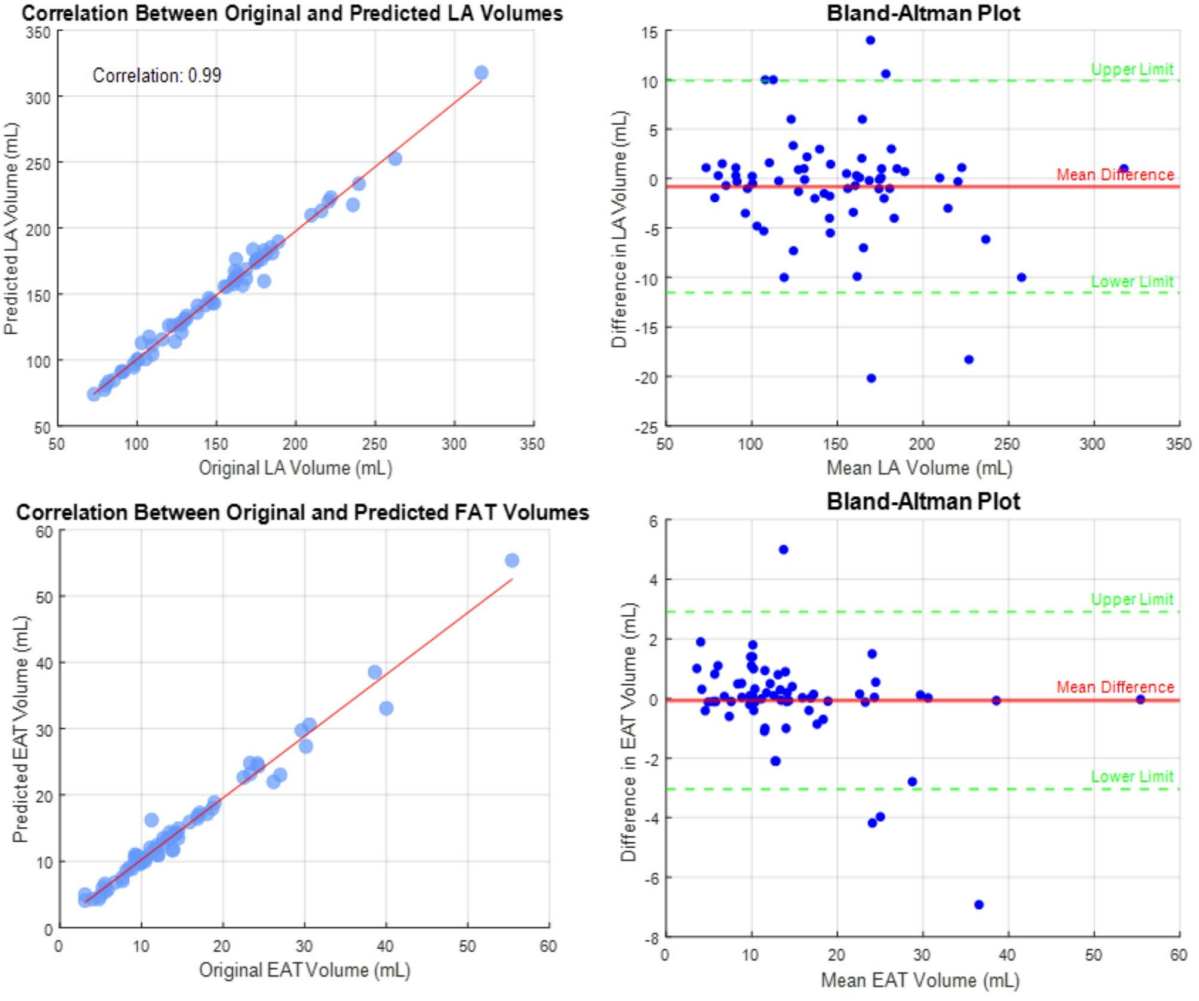
Comparison of Epicardial fat (EAT) and LA volumes between Ground Truth and Predicted Values from PoinUNET: Correlation and Bland–Altman Analysis.

The results of the correlation and Bland–Altman analyses for LA and EAT volume segmentation are shown in Fig. 6.

For LA volume, the correlation coefficient of 0.99 indicates an excellent linear relationship, demonstrating that PoinUNet segmentation closely aligns with the ground truth (Fig. 6). The Bland–Altman analysis reveals a mean difference of − 0.82 mL, suggesting a slight underestimation by PoinUNet. The limits of agreement, ranging from − 11.5 to 9.9 mL, highlight the variability between methods, with most differences falling within an acceptable range.

For EAT volume, the correlation coefficient of 0.99 reflects an equally excellent relationship. The mean difference of − 0.069 mL indicates an almost negligible underestimation by PoinUNet, while the limits of agreement (from − 3.0 to 2.9 mL) demonstrate the inter-method variability, with most differences falling within an acceptable range.

### Dependency of the method on type of AF and age

Understanding the influence of clinical factors such as AF type and patient age is essential for evaluating segmentation model performance. Table 5 presents the mean EAT and LA volumes for different types of atrial fibrillation (AF_Type) and age groups, comparing the ground truth with predictions made by the PoinUNet model. The results confirm that both AF type and age influence EAT and LA volumes.

**Table 5:** Dependency of the method on type of AF and age.

AF_Type/Age	Ground truth		PoinUNet	
	Mean_EAT	Mean_LA	Mean_EAT	Mean_LA
Paroxysmal atrial fibrillation (AF)	12.68 ± 1.5	113.42 ± 2.5	11.974 ± 5.5^a^	112.97 ± 40.2^a^
Persistent AF	10.308 ± 1.8	124.01 ± 10.9	10.282 ± 1.9^a^	125.67 ± 7.74^a^
Long-standing persistent AF	16.541 ± 3.5	158.95 ± 6.7	16.188 ± 3..3^a^	153.98 ± 11.5^a^
Permanent AF	22.739 ± 3.9	187.65 ± 18	22.537 ± 2.7^a^	185.76 ± 15.7^a^
‘ < 50 years’	7.7967 ± 1.5	130.4 ± 18.5	8.6267 ± 2.6^a^	133.87 ± 17^a^
‘50–60 years’	13.899 ± 3.4	120.8 ± 16.2	12.644 ± 3.9^a^	119.99 ± 12.1^a^
‘60–70 years’	15.134 ± 2.6	139.27 ± 10.5	14.843 ± 1.9^a^	139.38 ± 7.7^a^
‘ > 70 years’	17.852 ± 2.4	173.1 ± 14.6	17.936 ± 2.8^a^	169.54 ± 13.8^a^

The symbol ^a^ indicates that there were no statistically significant differences between PoinUNet and Ground Truth after applying T-test with Bonferroni correction.

Statistical analysis showed no significant differences between PoinUNet and ground truth measurements for EAT and LA volume across all AF subtypes and age groups, indicating strong agreement between the proposed method and manual annotations.

### Class imbalance in PoinUNet

PoinUNet addresses class imbalance using a combination of loss function adjustments and sampling strategies tailored for 3D medical image segmentation. Given the volumetric nature of the data, regions like EAT and the LA boundary occupy small portions of the overall volume. To mitigate this, patch-based sampling was employed to include underrepresented regions in each mini-batch, and balanced mini-batch sampling ensured an equal number of voxels from both classes. Data augmentations like rotation, scaling, and flipping were also applied to minority-class regions. For loss functions, PoinUNet utilized IDWH loss, which prioritizes boundary voxels, and weighted loss functions where the weight is inversely proportional to class prevalence. This helps to give minority-class voxels, like EAT regions, more influence during training.

Table 6 compares the effectiveness of the proposed loss function and the standard loss function in addressing class imbalance. Models trained without handling class imbalance (e.g., using cross-entropy loss) showed poorer performance, especially for EAT and LA segmentation. The combination of weighted Dice loss and augmentations improved results.

**Table 6:** Comparison of the proposed loss function and standard loss function for class imbalance handling on test set with mean LA volume 149.80 ± 4.3 mL and EAT volume 14.63 ± 1.9 mL.

Method	Dice score	LA volume	EAT volume
Cross-entropy loss	81.15 ± 3.5	150.45 ± 4.7	15.55 ± 1.7
Dice loss	83.00 ± 3.1	148.07 ± 4.5	14.37 ± 2.5
Dice loss + Cross-entropy loss	84.95 ± 2.8	149.97 ± 5.7	15.56 ± 2.6
The proposed loss	87.25 ± 2.8	149.84 ± 4.2	14.70 ± 1.3

## Discussion

In this study, we developed and validated a novel method for segmenting the LA wall and LA EAT from Dixon MRI data, leveraging a Poincaré layer to capture complex relational structures and global features. PoinUNet consistently outperformed both SwinUNETR and 3D UNet across key metrics such as Dice score, IoU, recall, and HD. Moreover, PoinUNet demonstrated a significant reduction in computation time, processing 3D MRI volumes in 90 seconds, compared to SwinUNETR 210 seconds for inference time. Two significant methodological contributions were introduced.

A hybrid representation combining both Euclidean and geometric structures (Euclidean and hyperbolic spaces) was integrated into the UNet architecture. The addition of a Poincaré layer improved voxel connections, aiding in the integration of complex target-related information and balancing local and global features. Importantly, the Poincaré layer can be easily incorporated into other networks, either alone or alongside convolution layers, making it a promising alternative to other complex shape regularization methods^26–28^.

The second contribution is the adaptive curvature for hyperbolic space, enhancing the model’s ability to handle complex data structures^22^. In hyperbolic space, distances expand exponentially from a reference point, making it ideal for modeling non-linear relationships and intricate spatial configurations. By using Poincaré layers, which operate in hyperbolic space, the model captures spatial relationships between the LA wall and EAT with greater precision than Euclidean methods, improving segmentation of LA EAT.

In medical image segmentation, various models have evolved to augment the capabilities of the UNet architecture. UNet, featuring an encoder-decoder structure, has been enriched with transformer blocks and convolutional neural networks to adeptly harness both global and local information. An illustrative instance is transformer-based UNet^29^, which seamlessly integrates transformers into the deep layers of the encoder, capturing extensive dependencies along with shallow CNN structures. In contrast, the UNETR model^30^ employs transformers as the entire encoder, strategically reducing computational complexity by placing them at the bottleneck, particularly advantageous in 3D tasks. Generally, the CNN-based methods like UNet tend to have over-segmentation problems, which may be due to the locality of the convolution operation^31^. The original SwinUNETR ^24^, which uses a patch-based approach with Transformer-based feature extraction, addresses some issues with preserving local features but still faces challenges. While the patch-based design helps to some extent, it does not fully resolve the problem of accurately segmenting EAT around LA. The SwinUNETR struggles to effectively separate the fat around the LA from other nearby organs, leading to less precise EAT segmentation. However, it performs well in segmenting the LA itself, indicating its capability in handling larger anatomical structures despite the difficulties with fine-grained local features.

The vision transformer adapter model incorporates hyperbolic embeddings and a spatial prior module, enhancing its ability to accurately delineate lesions^32^. By leveraging a vision transformer encoder, the model integrates spatial prior information through a spatial prior module, optimizing feature extraction. Furthermore, hyperbolic embeddings are utilized for pixel-level classification, capturing the underlying geometric structure of feature matrices. In contrast, PoinUNet combines Poincaré layers and convolutional layers to capture hierarchical relationships in hyperbolic space. While both models innovate in utilizing hyperbolic geometry, they differ in their architectural approach and application domain.

In medical imaging settings, computational efficiency is as crucial as segmentation accuracy, especially in time-sensitive or resource-limited environments. PoinUNet offers significantly faster inference time compared to SwinUNETR offers a distinct advantage, making it suitable for real-time applications such as point-of-care diagnostics. While SwinUNETR transformer-based architecture yields strong feature extraction, it demands more computational resources and time, limiting its practicality in such settings. PoinUNet, with its balanced design, achieves both high accuracy (Dice score of 0.87) and reduced computational load, making it more accessible for integration into healthcare systems where rapid and reliable segmentation is paramount.

The findings of high precision and Dice score, suggest that PoinUNet is well suited for clinical use, where minimizing false positives and ensuring boundary accuracy are more critical than maximizing recall. The slight reduction in recall is acceptable in this context, as higher precision ensures more reliable and accurate segmentations, particularly in medical imaging where false positives can lead to unnecessary interventions. The PoinUNet method for segmenting the LA wall and the LA EAT from cardiac MRI images has potential to facilitate expedited cardiac MRI analysis, personalized patient management, and further research endeavors. By automating the segmentation process, PoinUNet can enable efficient assessment of cardiac morphology, function and tissue characteristics adding pathophysiological aspects on the relationship between EAT accumulation/infiltration, atrial wall remodeling, and arrhythmogenesis in patients with AF.

The clinical relevance of PoinUNet for LA and EAT segmentation is further supported by studies showing significant associations between LA and EAT volumes and AF. Nakamori et al.^33^ highlight that LA-epicardial fat could be a therapeutic target for reducing AF burden, emphasizing the need for accurate quantification to identify patients at risk. Chahine et al. ^6^ demonstrate that LA EAT volume, measured using techniques like Dixon MRI, can predict AF recurrence post-ablation.

By means of enhanced LA EAT quantification, from rapid and reliable segmentation, PoinUNet has the potential to contribute to improved risk stratification and therapeutic strategies in patients with AF.

## Limitation and remaining challenges

The Dixon technique, which separates water and fat signals in MRI images, assists in visualizing cardiac structures such as the LA by isolating fat signals and improving the delineation of epicardial fat. However, LA segmentation remains challenging due to diverse atrial shapes and specific characteristics such as pulmonary vein arrangement. Despite advancements, variability in anatomy and imaging artifacts can hinder accuracy. Precise delineation is further complicated by surrounding structures. Addressing these challenges requires innovative approaches to enhance segmentation accuracy and reliability.

For EAT, the challenge is to distinguish artifacts from the boundary regions. Providing accurate LA wall segmentation is typically used to address this problem^34,35^. Here, Poincaré layer and relational attention were used to learn the spatial information of EAT around the wall. However, misclassification related to this issue may still occur due to limited training data. Another challenge is due to the varying intensity distributions of EAT, resulting in the data mismatch, further complicating the training process.

The dataset was sourced from a single site, and although the number of patients was small, it is rather typical for similar deep learning studies in medical image segmentation.

In the future, EAT detection may be enhanced by incorporating additional images as feature maps, including in-phase and out-of-phase images. Furthermore, adapting PoinUNet and applying hyperbolic training to SwinUNETR for EAT segmentation could reduce complexity, processing time, and the number of parameters. Additionally, integrating more skip connections in the bottleneck layer could enhance network accuracy. This can be achieved by employing various dilation rates, multi-scale downsampling, and exploring different hyperbolic spaces.

Manual segmentation was used for model validation, but we recognize its limitations. It is subjective, prone to variability, and time-consuming, which can hinder scalability. In the future, eliminating the need for manual segmentation and labels would be ideal. However, it is important to note that deep learning models currently rely on labeled data for training. Approaches like supervised learning or contrastive learning, which seek to reduce or eliminate the reliance on manual segmentation, could provide promising alternatives.

## Conclusion

PoinUNet, a novel method for segmenting the LA wall and LA EAT from Dixon MRI data, was developed and validated. By integrating Poincaré layers and adaptive hyperbolic curvature, PoinUNet effectively captures complex spatial relationships. PoinUNet outperformed state-of-the-art models like SwinUNETR and 3D UNet in both accuracy and computational efficiency, demonstrating its potential for real-time medical applications.

## Data Availability

The data underlying this article cannot be shared publicly due to limitations in ethical permits. Anonymized data may be shared on reasonable request to the corresponding author.
